# Effects of targeted phosphorylation site mutations in the DNA-PKcs phosphorylation domain on low and high LET radiation sensitivity

**DOI:** 10.3892/ol.2015.2974

**Published:** 2015-02-17

**Authors:** IAN M. CARTWRIGHT, JUSTIN J. BELL, JUNKO MAEDA, MATTHEW D. GENET, ASHLEY ROMERO, YOSHIHIRO FUJII, AKIRA FUJIMORI, HISASHI KITAMUTA, TADASHI KAMADA, DAVID J. CHEN, TAKAMITSU A. KATO

**Affiliations:** 1 Department of Environmental and Radiological Health Sciences, Colorado State University, Fort Collins, CO 80523, USA; 2 Department of Radiological Sciences, Ibaraki Prefectural University of Health Sciences, Inashiki, Ibaraki 300-0394, Japan; 3 Research Center for Charged Particle Therapy, International Open Laboratory, Inage, Chiba 263-8555, Japan; 4 Research, Development and Support Center, National Institute of Radiological Sciences, Inage, Chiba 263-8555, Japan; 5 Department of Radiation Oncology, University of Texas Southwestern Medical Center, Dallas, TX 75235, USA

**Keywords:** high linear energy transfer radiation, heavy ions, protons, DNA-protein kinase catalytic subunit

## Abstract

The present study investigated the effect of targeted mutations in the DNA-dependent protein kinase catalytic subunit and phosphorylation domains on the survival of cells in response to different qualities of ionizing radiation. Mutated Chinese hamster ovary V3 cells were exposed to 500 MeV/nucleon initial energy and 200 keV/μm monoenergetic Fe ions; 290 MeV/nucleon initial energy and average 50 keV/μm spread-out Bragg peak C ions; 70 MeV/nucleon initial energy and 1 keV/μm monoenergetic protons; and 0.663 MeV initial energy and 0.3 keV/μm Cs^137^ γ radiation. The results demonstrated that sensitivity to high linear energy transfer radiation is increased when both S2056 and T2609 clusters each contain a point mutation or multiple mutations are present in either cluster, whereas the phosphoinositide 3 kinase cluster only requires a single mutation to induce the sensitized phenotype of V3 cells. Additionally, the present study demonstrated that sensitivity to DNA cross-linking damage by cisplatin only requires a single mutation in one of the three clusters and that additional point mutations do not increase cell sensitivity.

## Introduction

DNA double-strand breaks (DSB) are the primary lesion induced by ionizing radiation and are most commonly associated with cell death (1). The cell has developed two major pathways of response to DSBs: Non-homologous end-joining (NHEJ) and homologous recombination (HR) (1). NHEJ is active throughout the cell cycle and is the repair pathway that deals with the majority of radiation-induced DSBs. NHEJ relies on a large protein complex, DNA-dependent protein kinase (DNA-PK), to bind to the ends of broken DNA and bring them together for direct ligation. DNA-PK is composed of the Ku70/80 end-binding proteins and the DNA-PK catalytic subunit (DNA-PKcs). The kinase activity of DNA-PK is directly dependent on the functional Ku subunit; however, the kinase activity is also affected by the loss of DNA-PKcs (2,3).

It appears that the phosphorylation activity of the DNA-PKcs contributes to the DSB repair capability of DNA-PK. DNA-PKcs not only have the ability to phosphorylate various NHEJ proteins, it is also able to autophosphorylate itself (4–6). DNA-PKcs has various phosphorylation sites throughout its protein structure, with the most critical sites located in the T2609 and the S2056 clusters (5–9). Site-directed mutants involving phosphorylation sites in the T2609 and S2056 clusters result in cell lines with various levels of radiosensitivity, ranging from a DNA-PKcs and Ku null phenotype to very mild sensitivity. This varying sensitivity was previously demonstrated in a synchronized G1 population of cells exposed to low linear energy transfer (LET) radiation Cs^137^ γ radiation (10).

High LET radiation is more effective at killing cells than low LET radiation. This increase in cell death can be observed as an increased relative biological effectiveness (RBE) when comparing the D_10_ value of cells exposed to 250 KeV X-rays or γ irradiation to the D_10_ value of cells exposed to high LET radiation. High LET radiation creates various types of complex DNA damage in small clusters within the DNA strand (11–14), including DBS, single-stranded breaks (SSBs) and base damage. Due to its complexity, the cell takes considerably longer to repair this high LET radiation-induced DNA damage (15,16). In contrast to the types of DNA damage caused by high and low LET radiation, cisplatin induces inter- and intrastrand cross-linking and DNA adducts (17–19). Cisplatin-induced damage is often resolved by the nucleotide excision repair pathway (20); however, occasionally, cisplatin can result in DSBs in dividing cells, and these DSBs require the NHEJ and HR pathways for complete repair (21).

The aim of the present study was to determine the relative sensitivity to high LET radiation of site-directed mutant cells, containing phosphorylatable residues in the T2609 cluster, S2056 cluster (19,20) and carboxyl-terminus phosphoinositide 3 kinase (PI3K) domain of DNA-PKcs. Furthermore, the current study expands upon a previous study conducted by Chen *et al* (8), which focused only on the sensitivity of these DNA-PKcs mutants to low LET radiation.

## Materials and methods

### Cell lines and cell culture

The present study utilized a wild-type Chinese hamster ovary (CHO) cell line (CHO10B2) provided by Dr. Joel Beford, Department of Evrionmental & Radiological Health Sciences, Colorado State University (Fort Collins, CO, USA); NHEJ-deficient xrs-5 (Ku80 mutated) and V3 cells; HR-deficient 51D1 (Rad51D mutated) cells provided by Dr. Larry Thompson, Biosciences and Biotechnology Division, Lawrence Livermore National Laboratory (Livermore, CA, USA); and 14 cell lines derived from DNA-PKcs null V3 cells with complementary human DNA-PKcs containing amino acid substitutions at specific positions (shown in Table I). Cells were cultured in minimal essential medium-α (Gibco Life Technologies, Indianapolis, IN, USA) supplemented with 10% fetal bovine serum (FBS; Sigma-Aldrich, St. Louis, MO, USA), and 1% penicillin, streptomycin and amphotericin B (Gibco Life Technologies, Carlsbad, CA, USA), and maintained at 37°C in a humidified atmosphere of 5% CO_2_ in air.

### Irradiation and cell treatment

Logarithmic phase cells were irradiated aerobically at room temperature. The radiation source was a JL Shepherd and Associates (San Fernando, CA, USA) irradiator that emitted Cs^137^ γ-rays at a rate of 2.5 Gy/min, and the cells were irradiated using accelerated Fe ions, C ions and protons at the National Institute of Radiation Sciences (Chiba, Japan). The LET of the radiation used were as followed: 500 MeV/nucleon initial energy and LET 200 keV/μm monoenergetic Fe ions; 290 MeV/nucleon initial energy and average LET 50 keV/μm spread-out Bragg peak (SOBP) C ions; 70 MeV/nucleon initial energy and LET 1 keV/μm monoenergetic protons; and, 0.663 MeV initial energy and LET 0.3 keV/μm Cs^137^ γ radiation. Additionally, the cells were exposed to various concentrations of cisplatin (3.3–33 μM) for 1 h prior to plating for the survival experiments. Survival curves were obtained by measuring the colony-forming ability of the irradiated cell populations. Briefly, post-irradiation, the cells were plated onto 60-mm plastic petri dishes and incubated for 7–10 days for colony formation. The dishes were then fixed with 100% ethanol and stained with 0.1% crystal violet solution. A colony with >50 cells was scored as a survivor.

### RBE

Prism 5™ software (GraphPad Software, Inc., La Jolla, CA, USA) was used to draw survival curves from the survival fraction obtained from the survival assay. This software was also used to obtain D_10_ values, the dose required to kill 90% of cells, and RBE values, by dividing the D_10_ values of the γ-ray exposure by the D_10_ values obtained various radiation exposures.

### Statistical analysis

Statistical comparison of the mean values was performed using a two tailed t-test. P<0.05 was considered to indicate a statistically significant difference. Error bars indicate the standard error of the means and confidence interval values were calculated using Prism 5™ software (GraphPad Software, Inc.). Additionally, variation amongst the cell lines was calculated using the D_10_ and mean values obtained from Prism 5™ software.

## Results

### Effect of site-specific mutations on sensitivity to low LET charged particle radiation (γ-rays and protons)

**Figure 1:**
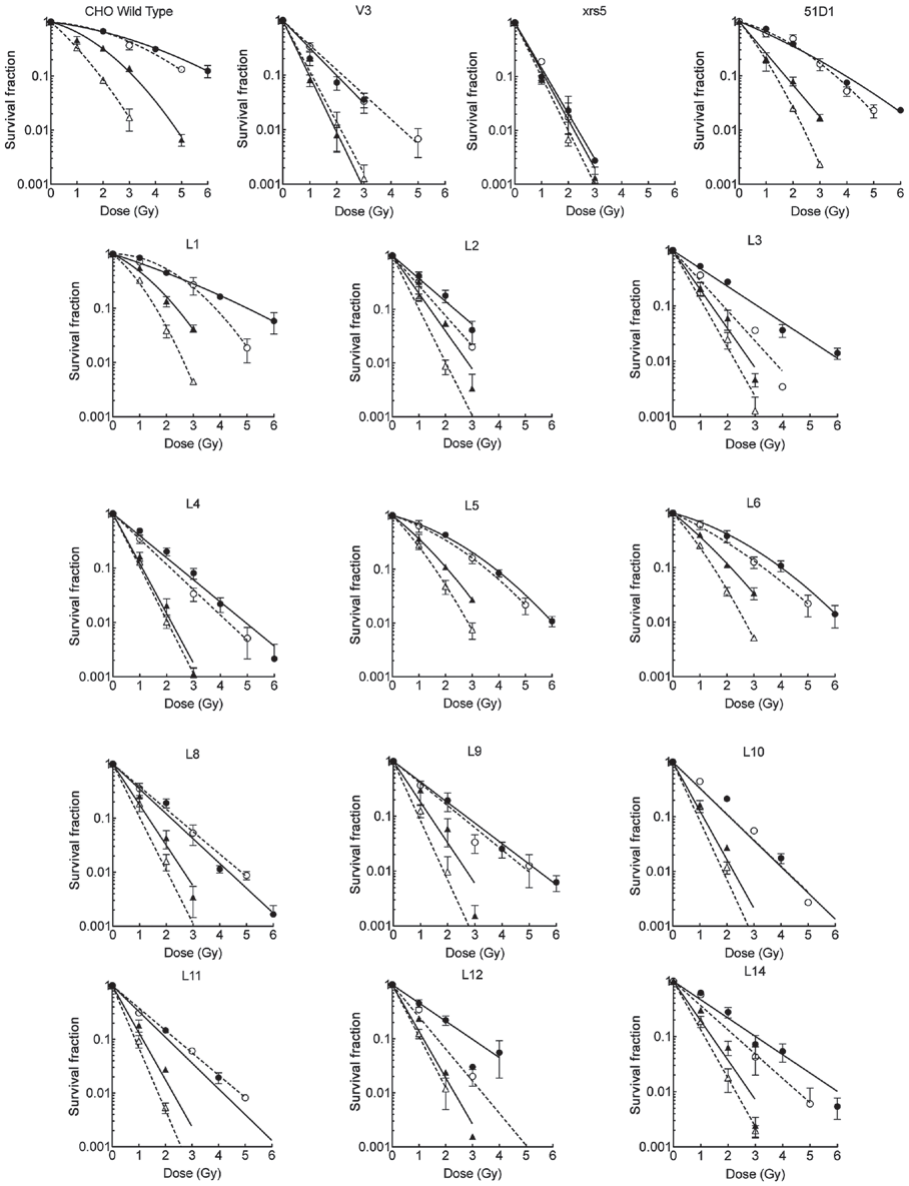
Survival curves of CHO mutant cell lines. CHO10B2, V3, xrs5, 51D1 and all L-series cells were exposed to 500 MeV/nucleon initial energy and 200 keV/μm monoenergetic Fe ions; 290 MeV/nucleon initial energy and average 50 keV/μm spread-out Bragg peak C ions; 70 MeV/nucleon initial energy and 1 keV/μm monoenergetic protons; and 0.663 MeV initial energy and 0.3 keV/μm Cs^137^ γ radiation. ●, γ radiation; ○, proton radiation; ▲, carbon ion radiation; △, iron ion radiation. Error bars represent the standard error of the mean. CHO, Chinese hamster ovary.

To investigate the role of DNA-PKcs in cellular sensitivity to low LET charged particles, the D_10_ values of the various DNA-PKcs mutants exposed to proton radiation were compared to the D_10_ values of the same cell lines exposed to γ-rays. Asychronized cells were exposed to 70 MeV/nucleon initial energy and LET 1 keV/μm protons or 0.663 MeV initial energy and LET 0.3 keV/μm of Cs^137^ γ-ray radiation, and were immediately sub-cultured and plated for colony formation assays. As shown in Table II, the D_10_ values obtained from the Prism5™ software indicated marginal variation between the DNA-PKcs mutants when exposed to proton radiation, similar to the values observed when the cells were exposed to γ irradiation. Furthermore, the xrs-5 cells demonstrated the highest sensitivity and the control cells demonstrated the highest resistance in these two groups. The majority of cell lines exhibited sensitivities similar to or more resistant than V3. The complete survival curves shown in Fig. 1 highlight the differing sensitivities between the DNA-PKcs mutant cell lines.

### Effect of site-specific mutations on sensitivity to high LET radiation (C and Fe ions)

Considering that the results for low LET charged particles were similar to those for low LET γ-rays, the effect of site specific mutations on the cells’ sensitivity to high LET radiation (Fe and C ions only) was investigated. Asychronized cells were exposed to 500 MeV/nucleon initial energy and 200 keV/μm monoenergetic Fe ions or 290 MeV/nucleon initial energy and average 50 keV/μm SOBP C ions, and were immediately subcultured and plated for colony formation assays. As Table II indicates, all DNA-PKcs mutants exhibited D_10_ values similar to the V3 cells, with the exception of L-5 and L-6. L-5 and L-6 demonstrated similar D_10_ values when compared with the L-1 cell line.

### Effect of site-specific mutations on RBE

Following calculation of the D_10_ values for each DNA-PKcs mutant and the control CHO10B2 cells at each exposure, the values were compared. As shown in Table III, the RBE values for all DNA-PKcs mutants are similar, demonstrating that the single point mutations do not increase the effectiveness of high LET radiation.

### Effect of site-specific mutations on the sensitivity of mutants to cisplatin

Cisplatin-induced DNA damage, unlike radiation-induced DNA damage, rarely causes DSBs. The various types of CHO cell were exposed to cisplatin, subcultured and plated for a survival assay. As shown in Table II, the DNA-PKcs mutants exhibit varying sensitivities to cisplatin. All the DNA-PKcs mutants investigated in the present study were more sensitive than the control CHO10B2 cells, however, no statistically significant difference between the sensitivity of the CHO10B2 and V3 cell lines was identified.

### Comparison between radiation and cisplatin sensitivity

**Figure 2:**
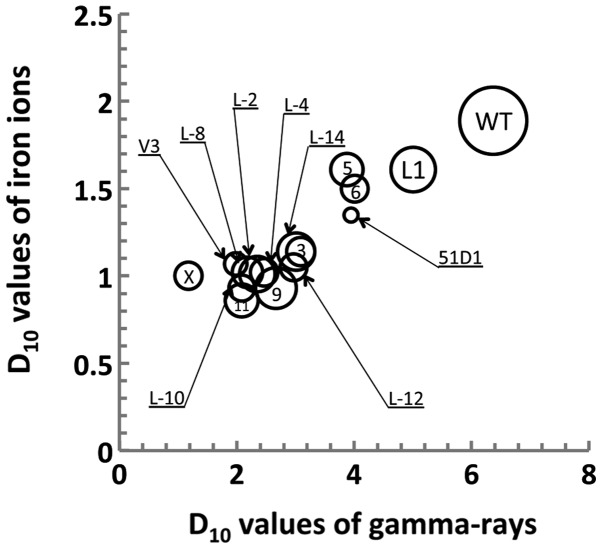
Comparison between γ-ray and Fe ion radiation, and cisplatin exposure D_10_ values using a bubble chart. Size of the circle corresponds to the D_10_ values of cisplatin. WT, Chinese hamster ovary 10B2 cells; X, xrs-5 cells.

To better understand the role of DNA-PKcs, the sensitivity of cisplatin exposure was compared with each of the low and high LET ionizing radiations used. The γ-ray D_10_ values were plotted on the X-axis, the Fe ion D_10_ values were plotted on the Y-axis and the D_10_ values of cisplatin were plotted in a bubble chart style, allowing the variance among each exposure group to be determined (Fig. 2).

As demonstrated in Fig. 2, radiation sensitivities between γ-ray and Fe ion radiation were correlated with the wild-type and DNA repair-deficient cell lines. However, the bubble chart indicated a lack of correlation between cisplatin and radiation sensitivity. L-11, one of the most sensitive mutants to γ-rays was also the most sensitive mutant to Fe ions. By contrast, L-6, which is one of the mutants most sensitive to cisplatin, is one of the mutants that is most resistant to γ irradiation.

## Discussion

The present study expands on a previous study, which demonstrated that low LET radiation damage to the DNA appears to only require a portion of the phosphorylation sites on the DNA-PKcs protein (10). Considering that high LET radiation produces complex damage to the DNA, this type of damage involves DSBs as well as adducts, cross-links and SSBs, all within a small region of DNA (11,12). In the current study, two types of low LET radiation, γ-rays and protons, and two types of high LET radiation, C and Fe ions, were utilized. C, Fe and proton radiation are all charged particles. As shown in Table II, L-5 and L-6 were the most resistant DNA-PKcs mutant cells to γ-ray and Fe ion radiation, however, L-5 and L-6 were also less resistant than the control and corrected cell lines (L-1) after γ-ray exposure. L-5 and L-6 each contain a single mutation in the S2056 and T2609 cluster, respectively. The L-4 cell line also contains these two point mutations, however, and was observed to exhibit increased sensitivity to γ-ray and Fe ion radiation. Thus, the L-4, L-3 and L-12 D_10_ data indicates that the V3 phenotype requires a mutation in the S2056 cluster and the T2609 cluster, or a complete loss of one of these clusters. Furthermore, the results obtained from the L-8, -9, -10 and -11 cell lines illustrate that the PI3K cluster is as important for repair of high LET-induced damage as it is for the repair of low LET-induced damage (10).

In addition to evaluating the role of specific phosphorylation DNA-PKcs sites in the repair of high LET radiation-induced DNA damage, the role of these phosphorylation sites in the repair of cisplatin-induced DNA damage was also investigated. As indicated in Fig. 2 and Table II, it appears that a single point mutation in the S2056, T2609 or PI3K cluster results in cisplatin sensitivity similar to that of the V3 null mutant. In contrast to the sensitivity to low and high LET-induced DNA damage, complete loss of a cluster or mutations in two clusters does not increase the sensitivity of the cell lines to cisplatin-induced DNA damage.

Finally, the correlation between the sensitivities of all the cell lines to low and high LET radiation-induced versus cisplatin-induced DNA damage was evaluated. A lack of correlation was identified, indicating that the contribution of cross-linking DNA damage, as a result of ionizing radiation, to cell death is minor. High LET radiation creates complex DNA damage consisting of DSB, SSBs, cross-linking and various other types of single nucleotide damage (11,13,14). Based on the D_10_ values of the L-5 and L-6 cell lines, single point mutations in the S2056 or T2609 clusters exhibited partial sensitivity to low LET radiation but appear to be insufficient for creating a V3 phenotype upon exposure to low LET radiation; however, L-5 and L-6 demonstrated a V3-like phenotype when exposed to cisplatin. This is in contrast to low LET-induced damage or cisplatin-induced damage, which requires a single mutation among three clusters to induce a V3 phenotype.

In conclusion, the present study demonstrated that the entire DNA-PKcs protein is required for repair of low LET radiation and cisplatin-induced DNA damage. However, a single mutation in the PI3K domain, multiple mutations within the S2056 or T2609 clusters, or two mutations in the S2056 and T2609 clusters, are required for the repair of high LET radiation-induced DNA damage. These results indicate that the interaction of two clusters may synergistically contribute to the repair of high LET radiation-induced DNA damage. However, further studies are required to investigate high LET-induced DNA damage and the associated molecular repair mechanisms.

## Figures and Tables

**Table I:** Cell lines derived from DNA-PKcs null V3 cells with human DNA-PKcs complementary DNA, containing amino acid substitutions at various positions in the DNA-PKcs constructs.

		Substituted domain														
		
		S2056 cluster					T2609 cluster						PI3K			
				
Cell line	Altered DNA-PKcs mutant	S2023	S2029	S2041	S2051	S2056	T2609	S2612	T2620	S2624	T2638	T2647	Y3715	L3750	K3752	D3921
L-1	Wild-type															
L-2	V3-7A					A	A	A	A	A	A	A				
L-3	V3-6A						A	A	A	A	A	A				
L-4	V3-2A					A	A									
L-5	V3-S2056A					A										
L-6	V3-T2609A						A									
L-8	V3-KA4														R	
L-9	V3-KB20													R	R	
L-10	V3-KC23^a^												Δ			
L-11	V3-KD51															N
L-12	V3-5A	A	A	A	A	A										
L-14	V3-3A						A				A	A				

a V3-KC23 mutant carries a frame-shift mutation at amino acid 3715 that resulted in truncation of the protein after 10 amino acids and loss of the entire PI3K kinase domain.

DNA-PKcs, DNA-dependent protein kinase catalytic subunit; PI3K, phosphoinositide 3 kinase; A, mutated alanine; R, mutated arginine; N, mutated asparagine; Δ, deletion.

**Table II:** D_10_ values of control and mutant cell lines to ionizing radiation and cisplatin.

	D_10_ (95% confidence interval)^a^				
	
Cell line	γ-rays, Gy	Proton, Gy	C ion, Gy	Fe ion, Gy	Cisplatin, μM
CHO10B2	6.37 (5.87–6.87)	5.31 (4.86–5.77)	3.16 (2.94–3.32)	1.89 (1.52–2.1)	29.29 (23.15–35.12)
XRS5	1.18 (1.05–1.31)	1.19 (0.90–1.46)	1.11 (0.94–1.28)	1.00 (0.95–1.05)	9.79 (8.85–10.70)
V3	1.98 (1.76–2.19)	2.21 (1.98–2.44)	0.97 (0.89–1.06)	1.07 (0.93–1.21)	11.86 (10.96–12.75)
51D1	3.95 (3.69–4.16)	3.57 (3.24–3.79)	1.71 (1.53–1.87)	1.35 (1.31–1.40)	6.15 (5.40–6.88)
L-1	5.01 (4.52–5.46)	3.82 (3.52–4.24)	2.38 (2.17–2.53)	1.61 (1.46–1.72)	19.41 (14.21–24.30)
L-2	2.35 (2.00–2.69)	1.79 (1.24–2.30)	1.42 (1.25–1.60)	1.01 (0.93–1.09)	15.45 (14.31–16.57)
L-3	3.09 (2.87–3.30)	1.84 (1.63–2.04)	1.42 (1.25–1.58)	1.14 (1.01–1.26)	12.31 (9.27–15.18)
L-4	2.47 (2.29–2.64)	2.15 (1.91–2.37)	1.09 (1.00–1.18)	1.02 (0.98–1.06)	11.81 (10.55–13.02)
L-5	3.88 (3.62–4.07)	3.54 (2.98–3.85)	2.08 (1.93–2.19)	1.61 (1.32–1.79)	14.09 (12.03–16.08)
L-6	4.01 (3.35–4.37)	3.35 (2.58–3.73)	2.14 (1.93–2.29)	1.50 (1.39–1.59)	11.67 (10.77–12.56)
L-8	2.18 (2.00–2.35)	2.40 (2.21–2.59)	1.33 (1.13–1.53)	1.02 (0.90–1.13)	13.27 (11.19–15.26)
L-9	2.67 (2.43–2.91)	2.48 (2.09–2.86)	1.35 (1.05–1.63)	0.93 (0.80–1.05)	17.76 (12.15–23.03)
L-10	2.09 (1.86–2.31)	2.10 (1.70–2.49)	1.12 (1.02–1.22)	0.93 (0.82–1.04)	11.30 (8.85–13.63)
L-11	2.08 (1.90–2.26)	2.40 (2.24–2.56)	1.14 (1.01–1.27)	0.86 (0.81–0.92)	14.37 (11.01–17.55)
L-12	2.97 (2.45–3.46)	1.68 (1.51–1.85)	1.16 (0.92–1.40)	1.05 (0.90–1.20)	11.71 (8.98–14.29)
L-14	3.01 (2.67–3.34)	2.27 (1.88–2.65)	1.40 (1.19–1.60)	1.14 (1.05–1.23)	16.17 (13.52–18.71)

a Calculated using Prism 5™.

**Table III:** Relative biological effectiveness^a^.

	Relative biological effectiveness			
	
Cell line	γ-rays	Protons	C ions	Fe ions
CHO10B2	1	1.20	2.02	3.37
XRS5	1	1.00	1.06	1.18
V3	1	0.90	2.04	1.85
51D1	1	1.11	2.31	2.93
L-1	1	1.31	2.11	3.11
L-2	1	1.31	1.65	2.32
L-3	1	1.68	2.18	2.71
L-4	1	1.15	2.27	2.42
L-5	1	1.10	1.87	2.41
L-6	1	1.20	1.87	2.67
L-8	1	0.91	1.64	2.14
L-9	1	1.08	1.98	2.87
L-10	1	1.00	1.87	2.25
L-11	1	0.87	1.82	2.42
L-12	1	1.77	2.56	2.83
L-14	1	1.33	2.15	2.64

a Relative biological effectiveness (RBE) was calculated using the following equation: RBE = (D_10_ γ) / (D_10_ test radiation).
